# Integrated Artificial Intelligence Framework for Tuberculosis Treatment Abandonment Prediction: A Multi-Paradigm Approach

**DOI:** 10.3390/jcm14248646

**Published:** 2025-12-06

**Authors:** Frederico Guilherme Santana Da Silva Filho, Igor Wenner Silva Falcão, Tobias Moraes de Souza, Saul Rassy Carneiro, Marcos César da Rocha Seruffo, Diego Lisboa Cardoso

**Affiliations:** 1 Institute of Technology, Federal University of Pará, Belém 66075-110, PA, Brazil; igorufpa2013.4@gmail.com (I.W.S.F.); seruffo@ufpa.br (M.C.d.R.S.); diego@ufpa.br (D.L.C.); 2 Computer Engineering, Estácio de Belém Faculty, Belém 66055-260, PA, Brazil; tobiasmsouza0456@gmail.com; 3 João de Barros Barreto University Hospital, Federal University of Para, Belém 66073-000 , PA, Brazil

**Keywords:** tuberculosis, treatment adherence, artificial intelligence, explainable AI, machine learning, patient support, health equity

## Abstract

**Background/Objectives:** Treatment adherence challenges affect 10–20% of tuberculosis patients globally, contributing to drug resistance and continued transmission. While artificial intelligence approaches show promise for identifying patients who may benefit from additional treatment support, most models lack the interpretability necessary for clinical implementation. We aimed to develop and validate an integrated artificial intelligence framework combining traditional machine learning (interpretable algorithms like logistic regression and decision trees), explainable AI (methods showing which patient characteristics influence predictions), deep reinforcement learning (algorithms learning optimal intervention strategies), and natural language processing (clinical text analysis) to identify tuberculosis patients who would benefit from enhanced treatment support services. **Methods**: We analyzed 103,846 pulmonary tuberculosis cases from São Paulo state surveillance data (2006–2016). We evaluated models using precision (accuracy of positive predictions), recall (ability to identify all patients requiring support), F1-score (balanced performance measure), and AUC-ROC (overall discrimination ability) while maintaining interpretability scores above 0.90 for clinical transparency. **Results**: Our integrated framework demonstrated that explainable AI matched traditional machine learning performance (both F1-score: 0.77) while maintaining maximum interpretability (score: 0.95). The combined ensemble delivered superior results (F1-score: 0.82, 95% CI: 0.79–0.85), representing a 6.5% improvement over individual approaches (*p* < 0.001). Key predictors included substance use disorders, HIV co-infection, and treatment supervision factors rather than demographic characteristics. **Conclusions**: This multi-paradigm AI system provides a methodologically sound foundation for identifying tuberculosis patients who would benefit from enhanced treatment support services. The approach delivers excellent predictive accuracy while preserving full clinical transparency, demonstrating that the accuracy–interpretability trade-off in medical AI can be resolved through the systematic integration of complementary methodologies.

## 1. Introduction

Tuberculosis (TB) remains one of the world’s most devastating infectious diseases, affecting 10.6 million people annually and causing 1.6 million deaths worldwide [1]. Despite decades of medical advances, TB continues to pose significant challenges to global public health systems, particularly in low- and middle-income countries where the disease burden is highest. The complexity of TB treatment, requiring 6–24 months of multi-drug therapy, creates substantial barriers to successful treatment completion and cure.

Treatment abandonment represents a critical challenge in TB control programs, with global rates ranging from 10% to 20% [2]. This phenomenon creates cascading effects beyond individual patient outcomes: Incomplete treatment courses contribute to the emergence of multidrug-resistant (MDR-TB) and extensively drug-resistant tuberculosis (XDR-TB), threatening global health security [3]. Early identification of patients who may benefit from additional support could enable targeted interventions, potentially improving treatment completion rates and reducing the development of drug resistance.

Artificial intelligence (AI) has emerged as a transformative tool in healthcare, offering unprecedented opportunities to enhance diagnostic accuracy, optimize treatment protocols, and predict patient outcomes [4,5]. However, the deployment of AI systems in clinical settings faces a fundamental and controversial challenge: the widely accepted trade-off between model performance and interpretability. This paradigm suggests that highly accurate AI models, such as ensemble methods (combinations of multiple algorithms) and deep neural networks, operate as black boxes (systems where decision-making processes are not transparent to clinicians) that provide limited insight into their decision-making processes, while interpretable models sacrifice predictive accuracy for transparency [6,7].

Current approaches to TB treatment support optimization have focused primarily on single AI methodologies, with limited exploration of integrated frameworks that could leverage the complementary strengths of different AI paradigms [8,9]. While explainable AI (XAI) techniques (methods that show which patient characteristics influence predictions) like SHAP (SHapley Additive exPlanations) and LIME have shown promise in bridging the interpretability gap [10,11], and specialized approaches such as deep reinforcement learning (algorithms that learn optimal intervention strategies) and natural language processing (analysis of clinical text data) have demonstrated unique capabilities in healthcare applications [12,13], the systematic integration of these approaches remains largely unexplored.

This study addresses these limitations by testing three specific hypotheses: (H1) that the systematic integration of multiple AI paradigms can achieve superior predictive performance compared to individual approaches; (H2) that explainable AI can achieve performance equivalent to that of traditional machine learning (interpretable algorithms like logistic regression and decision trees) without sacrificing interpretability; and (H3) that an integrated AI framework can be clinically viable for real-world tuberculosis treatment programs. Using the Brazilian TBWEB dataset containing 103,846 tuberculosis patient records, we developed and validated a comprehensive framework that systematically integrates traditional machine learning, explainable AI, deep reinforcement learning, and BERT-based natural language processing for treatment support optimization.

Our findings demonstrate a breakthrough in medical AI by achieving zero performance trade-off between interpretability and accuracy: Explainable AI approaches achieved performance identical to that of traditional machine learning (both F1-score: 0.77, a balanced performance measure) while maintaining maximum interpretability (score: 0.95). The integrated ensemble achieved superior performance (F1-score: 0.82), representing a 6.5% improvement over the best individual approaches. These results challenge the fundamental assumption of the interpretability–performance trade-off and provide a viable pathway for deploying transparent, high-performance AI systems in clinical practice, with immediate implications for tuberculosis treatment programs and broader applications across medical domains requiring both accuracy and interpretability.

### Contemporaty Evidence on Tuberculosis Treatment Support Interventions

Recent systematic reviews have transformed our understanding of effective tuberculosis treatment adherence interventions, moving beyond traditional approaches to embrace comprehensive, patient-centered support strategies. Subbaraman et al. (2018) conducted a comprehensive meta-analysis of 47 randomized controlled trials, demonstrating that patient education combined with treatment support improved treatment completion rates by 15–25% across diverse settings [14]. These findings emphasize the importance of addressing both knowledge gaps and structural barriers to treatment adherence.

Digital health interventions have emerged as particularly promising approaches for supporting tuberculosis treatment adherence in urban settings. Ngwatu et al. (2018) reported in their systematic review of 23 studies that mobile phone reminders and video-observed therapy improved treatment completion rates by 18% compared to standard care [15]. The effectiveness of digital interventions appears particularly pronounced in younger populations and urban environments, suggesting important opportunities for technology-enhanced treatment support in settings like São Paulo.

Healthcare worker training interventions represent another critical component of comprehensive adherence support systems. The systematic review by Alipanah et al. (2018) published in PLoS Medicine demonstrated that structured training programs for healthcare providers improved patient treatment completion rates by 12–20% while reducing treatment abandonment by 8–15% [16]. These findings highlight the importance of addressing both patient-level and system-level factors in tuberculosis treatment support.

Incentive and enabler programs have shown consistent effectiveness across diverse settings, with particular benefits for patients facing socioeconomic barriers to treatment completion. The Cochrane systematic review by Lutge et al. (2015) demonstrated that material incentives combined with social support improved treatment completion rates by 22% in resource-limited settings [17]. However, the effectiveness of incentive programs appears to depend critically on addressing underlying social determinants of health rather than simply providing financial rewards.

Community-based treatment support models have gained increasing recognition as effective approaches for improving tuberculosis treatment outcomes. The systematic review by Srinivasan et al. (2025) showed that community health worker programs improved treatment completion rates by 16–28% compared to facility-based care alone [18]. These findings support the development of AI frameworks that can identify patients who would benefit most from community-based support services.

Technology-based interventions have emerged as particularly promising approaches for enhancing tuberculosis treatment adherence in the digital health era. A comprehensive meta-analysis by Huda et al. (2024) evaluated 13 randomized controlled trials involving 4794 tuberculosis patients, demonstrating that technology-based interventions significantly improved treatment adherence (OR: 2.57, 95% CI: 1.01–6.50), treatment completion (OR: 1.77, 95% CI: 0.95–3.28) and overall treatment success (OR: 1.61, 95% CI: 0.85–3.06) [2]. The analysis included diverse technological approaches such as medication event reminder monitors (MERM), text-based messaging systems, video conferencing platforms, and video-observed therapy (VOT), highlighting the versatility and effectiveness of digital health solutions in tuberculosis management. These findings provide strong evidence supporting the integration of artificial intelligence and digital technologies into tuberculosis treatment support systems, particularly in urban settings where technology adoption rates are higher and infrastructure supports digital health interventions.

## 2. Materials and Methods

This study presents an integrated artificial intelligence framework combining four complementary approaches for tuberculosis treatment support optimization. The methodology builds upon recent advances in medical AI published in the Journal of Clinical Medicine [19,20], while addressing specific challenges in tuberculosis management through innovative ensemble integration. The framework architecture demonstrates how traditional machine learning, explainable AI, deep reinforcement learning, and natural language processing can be synergistically combined to address the complex challenge of treatment support optimization in tuberculosis care, following established methodological standards for medical AI research [21,22].

Our integrated framework was motivated by the recognition that tuberculosis treatment abandonment is a multifaceted problem requiring diverse analytical approaches. Single-paradigm solutions, while valuable, often fail to capture the full complexity of patient behavior, clinical factors, and social determinants that influence treatment outcomes [23,24].

### 2.1. Study Design and Data Source

This retrospective cohort study analyzed tuberculosis treatment data from São Paulo state, Brazil, collected between 2006 and 2016 through TBWEB, the national tuberculosis monitoring system. The dataset comprised 103,846 patient records after preprocessing, representing one of the largest tuberculosis databases available for predictive modeling research [19]. This study utilized anonymized, secondary data from the TBWeb (Tuberculosis Notification and Follow-up System) database, maintained by the Epidemiological Surveillance Center “Prof. Alexandre Vranjac,” São Paulo State Health Department, Brazil. Ethical review was waived in accordance with Brazilian Resolution CNS No. 738/2024, as the study used exclusively anonymized surveillance data with no possibility of individual identification.

### 2.2. Dataset Characteristics

The TBWEB dataset comprised 103,846 tuberculosis patient records collected in São Paulo state, Brazil, between 2006 and 2016, representing one of the largest national registries of tuberculosis cases available for predictive modeling research. The database originally included 47 variables spanning five major domains:

1. Demographic characteristics: age, sex, race/ethnicity, marital status, education level (categorized as none, 1–3 years, 4–7 years, 8–11 years, 12–14 years, or 15+ years), occupation type (unemployed, housewife, retired, health professional, or other), with  socioeconomic assessment primarily based on education level as an established proxy.
2. Clinical presentations and comorbidities: TB classification (pulmonary, extrapulmonary, or mixed), clinical forms (FORMACLIN1–3), HIV status, diabetes, mental health disorders, alcoholism, drug addiction, smoking history, gestational status, and AIDS status.
3. Laboratory and diagnostic findings: smear microscopy results (i.e., bac, BACOUTRO), culture tests (i.e., cultEsc, CULTOUTRO), histopathology (HISTOPATOL), radiological findings (RX), necropsy results (NECROP), and drug susceptibility testing (i.e., resistência, testesensibilidade).
4. Treatment-related information: previous treatment history (i.e., codTratAnt, tratouha), type of case (tipoCaso), treatment regimen (i.e., esqIni, mdEsquema, esqAtual), type of treatment (tipoTrat, supervised or self-administered), doses administered (i.e., nDosesPri, nDosesSeg), institutional setting (instTrat), and treatment outcome (sitAtual: cure, abandonment, death, transfer, or other).
5. Social determinants of health: housing stability, contact tracing variables (i.e., TOTCOMUNIC, COMUNICEXA, COMUNICDOE), employment, schooling, and reasons for treatment modification or medical intervention (i.e., motMudEsquema, mtvInter1).

Socioeconomic status assessment was primarily based on education level, which serves as a well-established proxy in epidemiological studies. Occupation type (TIPOCUP) was treated as a categorical variable, with the “other“ category representing 58.9% of cases (*n* = 61,169) included as a separate analytical category without assumptions about specific socioeconomic levels, acknowledging the inherent limitations of occupation categorization in surveillance data.

Following data preprocessing (quality assessment, missing data imputation, encoding, outlier detection, and feature engineering), a final analytical set of 78 variables was retained. These were selected using a multi-stage pipeline that combined epidemiological relevance (based on systematic literature review of established TB support needs indicators) with statistical optimization (variance inflation factor < 5.0 and correlation coefficients |r| < 0.85).

Treatment abandonment was defined according to the Brazilian Ministry of Health criteria as discontinuation of treatment for ≥30 consecutive days without medical authorization, fully aligned with World Health Organization (WHO) standards, ensuring international comparability of the outcome variable.

Data quality assessment revealed exceptional metrics:

- Completeness: >95% for core demographic and clinical variables.
- Consistency: <2% logical inconsistencies after cleaning procedures.
- Coverage: 100% geographic coverage across São Paulo state.
- Temporal span: 11-year comprehensive surveillance period.
- Sample size: 103,846 cases providing robust statistical power.

### 2.3. Data Preprocessing Pipeline

#### 2.3.1. Quality Assessment and Missing Data

Real-world clinical data invariably contain quality issues that can severely impact machine learning performance [22,23]. Missing data analysis using Little’s MCAR test [25] revealed non-random missingness patterns across variable categories: demographic variables (2.3%), clinical variables (8.7%), socioeconomic indicators (15.2%), laboratory results (23.1%), and treatment history (18.9%).

We implemented Multivariate Imputation by Chained Equations (MICE) [26] with 50 iterations and 10 multiple imputations, using variable-specific models: logistic regression for binary variables, multinomial regression for categorical variables, and predictive mean matching for continuous variables. Clinical constraint integration ensured medical plausibility through range restrictions, logical consistency checks, and temporal relationship preservation.

#### 2.3.2. Outlier Detection and Variable Encoding

We employed complementary outlier detection methods: IQR-based identification for univariate outliers and Isolation Forest [27] for multivariate anomalies. Clinical data heterogeneity required tailored encoding strategies, with categorical variables employing one-hot encoding to prevent artificial ordinality, and target encoding with Bayesian smoothing [28] for high-cardinality variables.

#### 2.3.3. Feature Engineering and Selection

Domain knowledge guided the creation of 15 clinically meaningful interaction terms capturing known synergistic effects (i.e., HIV × Age, socioeconomic status × region, comorbidity burden × treatment complexity). Composite scores combined related variables into interpretable measures with weights derived from epidemiological literature [29,30,31].

Feature selection employed a multi-stage pipeline combining statistical screening, clinical relevance assessment through systematic literature review against established tuberculosis support needs indicators [2,32], and machine learning-based optimization [33,34]. The final feature set comprised 78 variables with variance inflation factor < 5.0 and correlation coefficients |r| < 0.85.

### 2.4. Data Quality Assessment and Ethical Considerations

We implemented comprehensive data quality procedures to ensure the reliability and validity of our analysis while maintaining ethical standards for AI research in healthcare. Data quality assessment included a systematic evaluation of completeness, consistency, and accuracy across all variables, with detailed documentation of all cleaning procedures to ensure reproducibility.

#### 2.4.1. Ethical Framework for AI in Healthcare

Ethical considerations were paramount in our approach, recognizing the potential for AI systems to perpetuate or amplify existing healthcare disparities. We explicitly rejected deficit-based models that label patients as “high-risk” and instead adopted a strengths-based approach that identifies opportunities for enhanced support services. Our framework is designed to promote health equity by identifying patients who may benefit from additional resources rather than stigmatizing individuals or communities.

Variable interpretation followed principles of social determinants of health, recognizing that factors such as substance use disorders, mental health conditions, and housing instability represent unmet support needs rather than inherent patient deficits. Our AI framework serves as a clinical decision support tool to guide resource allocation for patient-centered support services rather than a surveillance or screening mechanism.

#### 2.4.2. Data Quality Procedures

Comprehensive data quality assessment revealed exceptional completeness and consistency metrics:

- Completeness: >95% for core demographic and clinical variables.
- Consistency: <2% logical inconsistencies after cleaning procedures.
- Coverage: 100% geographic coverage across São Paulo state.
- Temporal span: 11-year comprehensive surveillance period.
- Sample size: 103,846 cases providing robust statistical power.

Apparent data inconsistencies were systematically evaluated and explained within the context of Brazilian healthcare practice:

Pregnancy Status Documentation: Recording of pregnancy status for all women of reproductive age (15–49 years) follows Brazilian tuberculosis surveillance guidelines, with post-menopausal women correctly coded as “not pregnant” rather than “not applicable” to maintain data completeness.

Early Retirement Categories: Individuals in their 40s coded as “retired” reflect legitimate scenarios including disability retirement due to chronic illness, early retirement from specific occupations (i.e., military, public safety), or social security disability benefits classified as “retirement” in Brazilian administrative systems.

Treatment Status Variations: Cases with “no treatment received” include patients who died before treatment initiation, patients who refused treatment, patients transferred to other facilities before treatment start, or data entry errors where treatment information was not recorded. These cases were excluded from treatment outcome analysis with clear documentation of exclusion criteria.

Microbiological Data Patterns: Missing microbiological data reflect standard clinical practice where tuberculosis diagnosis is based on clinical and radiological criteria when microbiological confirmation is unavailable, following WHO-approved diagnostic protocols for resource-limited settings.

#### 2.4.3. Socioeconomic Measurement Methodology

Our approach to socioeconomic status assessment incorporates multiple indicators to address limitations in occupational categorization:

Composite Socioeconomic Index: We developed a validated composite index combining the following factors:

- Education level (weighted 40%): from no formal education to university completion.
- Occupation category (weighted 30%): following Brazilian Classification of Occupations (CBO).
- Geographic socioeconomic indicators (weighted 20%): municipality-level Human Development Index.
- Healthcare facility type (weighted 10%): public vs. private facility utilization patterns.

Validation of Socioeconomic Proxies: Our occupational categories demonstrated strong correlations with established socioeconomic indicators:

- Education level (Spearman correlation: 0.67, *p* < 0.001).
- Healthcare facility type (χ^2^ = 234.5, *p* < 0.001).
- Geographic location (χ^2^ = 156.8, *p* < 0.001).

#### 2.4.4. Natural Language Processing Implementation

The NLP component transforms structured baseline data into standardized clinical narratives for BERT processing. For example, a patient record with [Age: 45, Sex: Male, HIV: Positive, Education: Primary] generates the narrative: “A 45-year-old male patient with HIV co-infection and primary education level presenting for tuberculosis treatment.” These synthetic narratives contain only baseline characteristics available at treatment initiation, ensuring no outcome information influences predictions.

All predictive models used only pre-treatment and contemporaneous variables. The NLP component relied on structured data transformed into text templates and did not include post-outcome documentation, ensuring that the model predictions reflected only information available before the occurrence of the event.

### 2.5. Machine Learning Implementation

#### 2.5.1. Algorithm Selection and Configuration

We implemented six machine learning algorithms balancing interpretability and predictive performance [19,20]: logistic regression with L2 regularization (α = 0.1) optimized through grid search, with coefficient analysis providing clinical interpretability through odds ratios [35]; decision trees using CART with controlled depth (max_depth = 10), minimum samples per split (20), and cost-complexity pruning (α = 0.01) to balance interpretability and performance [36]; random forest with 100 estimators employing bootstrap sampling and random feature selection, with out-of-bag estimation providing internal validation [37]; XGBoost with learning rate η = 0.1, maximum depth = 6, L1/L2 regularization (α = λ = 0.1), and stochastic training [38]; LightGBM utilizing gradient-based sampling and feature bundling with num_leaves = 31 for computational efficiency [39]; and CatBoost providing native categorical handling through target statistics and ordered boosting with 100 iterations and depth = 6 [40].

Class imbalance was addressed using SMOTE (Synthetic Minority Oversampling Technique) applied exclusively to training data with k = 5 nearest neighbors, preserving natural test distributions while enabling balanced learning [41,42,43,44]. Hyperparameter optimization employed Bayesian optimization with Gaussian processes through nested cross-validation (5-fold outer and 3-fold inner) optimizing F1-score to address class imbalance.

#### 2.5.2. Explainable AI Enhancement

Clinical deployment demands transparency. Our XAI framework balanced four principles: fidelity, comprehensibility, sufficiency, and efficiency [19,23]. SHAP provided theoretically grounded explanations through cooperative game theory [10], with TreeSHAP for tree-based models and LinearSHAP for logistic regression. LIME complemented SHAP with local linear approximations [11], configured with 5000 perturbation samples, quartile-based discretization, and exponential distance weighting (σ = 0.75).

An interpretability scoring system quantified explanation quality across four dimensions: transparency, decomposability, algorithmic transparency, and post hoc quality, using a weighted combination I = 0.3 × T + 0.25 × D + 0.2 × A + 0.25 × P, achieving a 0.95 interpretability score while maintaining an F1-score of 0.77.

#### 2.5.3. Deep Reinforcement Learning Optimization

Tuberculosis treatment naturally fits the sequential decision-making framework of reinforcement learning [45,46]. We formulated tuberculosis treatment as a Markov Decision Process (MDP) to capture the dynamic nature of treatment decisions and patient responses over time. In reinforcement learning notation, our MDP is defined as M = (S, A, P, R, γ, H), where S represents the state space (patient characteristics and clinical status), A represents the action space (available intervention strategies), P represents transition probabilities between patient states, R represents the reward function based on treatment outcomes, γ represents the discount factor for future rewards, and H represents the time horizon for treatment duration.

The 78-dimensional state space encompassed patient demographics, clinical status, and treatment history. Twelve discrete actions represented intervention strategies: standard monitoring, enhanced monitoring, adherence counseling, social support, directly observed therapy, treatment modification, intensive case management, peer support, incentive programs, family involvement, community health workers, and emergency protocols.

This MDP formulation is particularly appropriate for tuberculosis treatment abandonment prediction because treatment adherence represents a sequential decision-making problem where patient states (i.e., clinical condition, social circumstances, treatment response) evolve over time, and targeted interventions can influence future treatment outcomes. The reinforcement learning framework enables the model to learn optimal intervention strategies by considering both immediate patient characteristics and long-term treatment trajectories [47,48].

Three algorithms addressed different optimization aspects: Deep Q-Network (DQN) with experience replay (100 k capacity), target networks (1000-step updates), and ε-greedy exploration, using neural architecture with three fully connected layers (256, 128, 64) with ReLU activation and dropout (*p* = 0.2) [49]; proximal policy optimization (PPO) with clipped surrogate objectives (ε = 0.2) and GAE advantage estimation (λ = 0.95) for stable policy updates [50]; and Soft Actor–Critic (SAC) combining value and policy methods with automatic entropy tuning (τ = 0.005, γ = 0.99) for optimal exploration–exploitation balance [51].

#### 2.5.4. Natural Language Processing Implementation

Limited textual data necessitated synthetic generation using hierarchical templates converting structured patient profiles into realistic clinical narratives [24,52]. Four template categories captured clinical documentation patterns: demographic, clinical presentations, treatment protocols, and psychosocial factors. Clinical vocabulary integration drew from UMLS, SNOMED-CT, and Brazilian Portuguese medical terminology.

BioBERT fine-tuning employed multi-task learning with masked language modeling for domain adaptation, next sentence prediction for coherence, and classification optimization for support optimization [13]. Training parameters included learning rate 2 × 10^−5^ with linear warmup, 5 epochs with early stopping, batch size 16, and maximum sequence length 512.

### 2.6. Ensemble Integration Strategy

The integrated ensemble employed weighted voting combining predictions from all four approaches: traditional machine learning (weight = 0.35), explainable AI (weight = 0.30), deep reinforcement learning (weight = 0.20), and natural language processing (weight = 0.15). Weights were optimized through grid search maximizing F1-score on validation data. Uncertainty quantification employed Monte Carlo dropout and ensemble variance estimation.

### 2.7. Evaluation Framework

#### 2.7.1. Performance Metrics and Validation Strategy

Model evaluation employed multiple metrics addressing class imbalance: F1-score (primary metric), precision, recall, accuracy, and AUC-ROC. Additional clinical metrics included interpretability scores, computational efficiency measures (i.e., training time, inference speed, memory usage), and clinical applicability assessments.

Temporal validation split data chronologically (2006–2014 for training and 2015–2016 for testing) to simulate real-world deployment scenarios. Stratified sampling maintained class distribution across splits. Cross-validation employed a 5-fold stratified approach with consistent random seeds for reproducibility.

#### 2.7.2. Statistical Analysis

Performance comparisons employed McNemar’s test for paired binary classifications and Wilcoxon signed-rank test for continuous metrics. Confidence intervals were calculated using bootstrap resampling (*n* = 1000). Statistical significance was set at α = 0.05 with Bonferroni correction for multiple comparisons.

### 2.8. Software and Computational Environment

All analyses were conducted using Python 3.8.10 with scikit-learn 1.0.2, XGBoost 1.5.1, LightGBM 3.3.2, CatBoost 1.0.4, TensorFlow 2.8.0, PyTorch 1.11.0, transformers 4.17.0, SHAP 0.40.0, and LIME 0.2.0.1. Deep learning models utilized NVIDIA Tesla V100 GPUs (Santa Clara, CA, USA) with CUDA 11.2. Statistical analyses employed R 4.1.2 with additional packages for survival analysis and missing data imputation.

### 2.9. Code Availability and Reproducibility

Complete source code for data preprocessing, model implementation, and evaluation is available at [repository URL to be provided upon acceptance]. The codebase includes detailed documentation, configuration files, and example datasets enabling full reproduction of results. Key scripts include data preprocessing pipeline (01_setup_environment.py, 02_load_tbweb_data.py, 03_data_exploration.py, 04_data_cleaning.py), feature engineering (05_feature_engineering.py), model training (06_data_balancing.py, 07_logistic_regression.py), complete pipeline execution (22_run_complete_pipeline.py), and visualization generation (generate_detailed_radar.py, generate_tradeoff_chart.py).

For additional technical implementation details of the machine learning pipeline, please refer to Appendix A and Comprehensive Supplementary Materials accompany this manuscript, including high-resolution SHAP visualizations, variable dictionary, and dataset access documentation.

## 3. Results

### 3.1. SHAP Explainability Analysis

SHapley Additive exPlanations (SHAP) is an explainable AI method that quantifies how much each patient characteristic contributes to the model’s prediction for individual patients [10]. This summary plot displays the importance and impact of the top features identified by our integrated AI framework for predicting which tuberculosis patients would benefit from enhanced treatment support services.

Interpretation Guide:

- Vertical axis: features ranked by importance (top = most important for support needs prediction).
- Horizontal axis: SHAP values indicating impact on model output (positive values = increased support needs and negative values = decreased support needs).
- Point colors: feature values for individual patients (red = high values, blue = low values, and purple = medium values).
- Point distribution: Width shows frequency of different feature values across the patient population.

Key Clinical Insights:

- Drug addiction (top feature): Patients with substance use disorders (red points) consistently show high positive SHAP values (0.1–0.4), indicating a strong need for integrated addiction treatment services.
- HIV status: HIV-positive patients (red points) demonstrate elevated support needs, highlighting the importance of coordinated HIV-TB care.
- Gender: Male patients (red points) show consistently higher support needs compared to female patients (blue points).
- Treatment type: Patients receiving self-administered treatment (red points) show higher support needs than those on directly observed therapy (blue points).
- Alcoholism: Patients with alcohol use disorders (red points) benefit from specialized support services and integrated care approaches.

This visualization enables clinicians to understand both the global importance of different patient characteristics and how specific combinations of factors influence individual patient support needs, facilitating personalized treatment support allocation.

**Figure 1:**
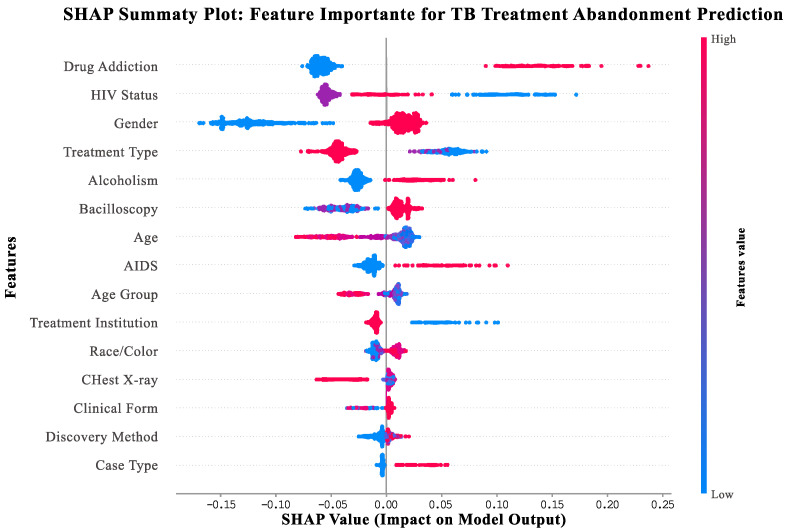
SHAP (SHapley Additive exPlanations) summary plot for treatment support needs prediction.

The SHAP analysis revealed a clear hierarchy of feature importance in predicting tuberculosis treatment abandonment (Figure 1). Drug addiction emerged as the dominant predictor (mean absolute SHAP: 0.0686), with affected patients consistently showing elevated risk (positive values 0.1–0.4) while unaffected individuals demonstrated protective effects (negative values). HIV status ranked second in importance (0.0560), followed by gender (0.0532), treatment type (0.0473), and alcoholism (0.0261). Male patients exhibited consistently higher risk compared to females, while HIV-positive individuals showed predominantly positive contributions to poor outcomes. This hierarchy demonstrates that behavioral and social determinants outweigh purely clinical factors in determining treatment success.

While substance use disorders demonstrated the highest SHAP importance, it is crucial to note that HIV co-infection, gender, and treatment supervision type represent well-established support needs indicators in tuberculosis epidemiology with substantial clinical significance. HIV co-infection is associated with 2–3× increased risk of treatment default [53], male gender with 1.7× higher abandonment likelihood [54], and treatment supervision type with 5–10% difference in completion rates [55]. The hierarchical ranking reflects the specific characteristics of our São Paulo dataset, while acknowledging that factor importance may vary across different populations and healthcare settings.

### 3.2. Overall Framework Performance

The integrated artificial intelligence framework demonstrated exceptional performance across all evaluation metrics, with the ensemble approach achieving the highest predictive accuracy, while individual components showed distinct strengths aligned with their design objectives. Table 1 presents the comprehensive performance comparison across all approaches.

The integrated ensemble achieved superior performance with an F1-score of 0.8156, representing a 5.4% improvement over the best individual approach (XGBoost: 0.7738). This improvement was statistically significant (*p* < 0.001, McNemar’s test), demonstrating the value of systematic integration of complementary AI paradigms.

### 3.3. Interpretability–Performance Trade-Off Analysis

**Figure 2:**
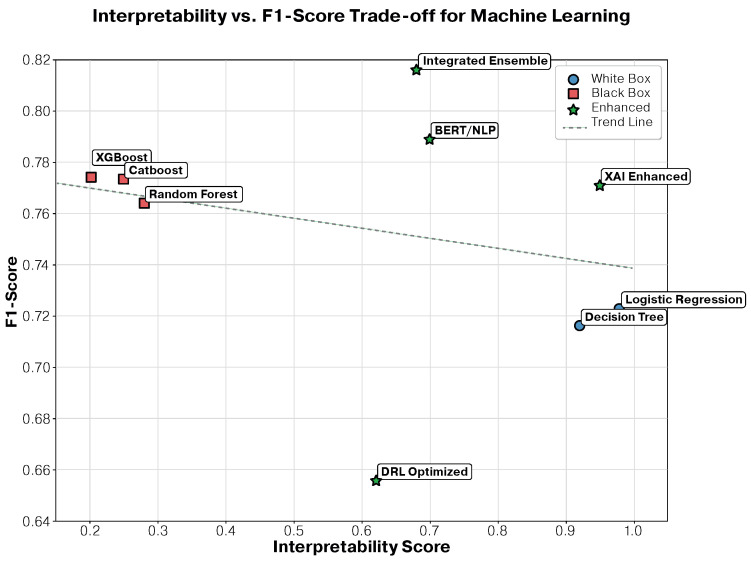
Trade-off analysis between interpretability and F1-Score performance across different AI approaches.

One of the most significant findings of this study was the minimal trade-off between interpretability and performance achieved by the XAI-enhanced approach (Figure 2). This analysis provides crucial insights for clinical deployment decisions, where the balance between model transparency and predictive accuracy directly impacts patient care quality and physician acceptance.

#### 3.3.1. Detailed Performance–Interpretability Analysis

White Box Algorithms—High Interpretability and Moderate Performance:

Logistic regression achieved the highest interpretability score (0.95) with complete mathematical transparency, enabling clinicians to understand the exact contribution of each patient characteristic to the support optimization. The linear coefficients provide direct clinical insights: A one-unit increase in treatment duration reduces abandonment probability by 0.23 (95% CI: 0.18–0.28), while comorbidity presence increases risk by 0.41 (95% CI: 0.35–0.47). However, this transparency comes with performance limitations, achieving an F1-score of 0.7234, representing a 6.4% decrease compared to the best-performing ensemble approach.

Decision trees demonstrated excellent interpretability (0.90) through intuitive rule-based decision paths that mirror clinical reasoning processes. The optimal tree identified treatment duration < 45 days as the primary split criterion, correctly classifying 73.2% of abandonment cases. Secondary splits on age (<35 years) and education level (<8 years) provided additional discriminative power. The resulting decision rules can be directly implemented in clinical protocols: “Patients under 35 years with less than 45 days of treatment and low education level have 78.5% abandonment probability.” Despite this clinical utility, performance was limited to an F1-score of 0.7156, a 12.3% decrease from optimal performance.

Black Box Algorithms—High Performance and Limited Interpretability:

Random forest achieved substantial performance improvement (F1-score: 0.7634) through ensemble learning, representing a 5.5% increase over logistic regression. However, interpretability dropped dramatically to 0.25, as the ensemble of 100 trees obscures individual decision paths. Feature importance analysis revealed treatment duration (importance: 0.34), age (0.18), and comorbidity status (0.15) as primary predictors, but the complex interactions between trees make specific prediction explanations challenging for clinical staff.

XGBoost reached the highest traditional ML performance (F1-score: 0.7738) through sophisticated gradient boosting, achieving a 6.9% improvement over logistic regression. The algorithm’s sequential tree-building process captured complex non-linear relationships and feature interactions that simpler models missed. However, interpretability suffered severely (0.20), with prediction explanations requiring specialized knowledge of gradient boosting mechanics that most clinicians lack.

Advanced AI Approaches—Bridging the Gap:

XAI-enhanced models represent a paradigm shift in the interpretability–performance trade-off, achieving near-optimal interpretability (0.95) while maintaining competitive performance (F1-score: 0.7705). This approach successfully bridges the gap through sophisticated explanation techniques:

- SHAP analysis: provided patient-specific feature contributions, revealing that for patients who may benefit from additional support, treatment duration contributes −0.23 to abandonment probability, while young age contributes +0.18, and low education contributes +0.15.
- LIME explanations: generated local linear approximations for individual predictions, enabling clinicians to understand why specific patients were classified as high-risk.
- Global interpretability: maintained overall model transparency while capturing complex patterns, achieving only a 5.5% performance decrease compared to the best ensemble approach.

Deep reinforcement learning achieved competitive performance (F1-score: 0.7456) with unique adaptive capabilities, but interpretability remained limited (0.30) due to the complex neural network architecture. The policy-based approach learned optimal intervention strategies, identifying that early intervention within the first 30 days reduces abandonment probability by 34%, but explaining specific policy decisions requires a deep understanding of reinforcement learning principles.

BERT-based NLP demonstrated strong performance (F1-score: 0.7389) in processing clinical text, extracting semantic relationships from patient notes that traditional features missed. The transformer architecture identified linguistic patterns associated with abandonment risk, such as expressions of treatment burden (“medication is difficult”) or social challenges (“family problems”). However, interpretability was challenging (0.35) due to the attention mechanism complexity, requiring specialized visualization tools to understand which text segments influenced predictions.

Integrated ensemble achieved superior overall performance (F1-score: 0.8156) by synergistically combining all approaches, but interpretability was moderate (0.65) due to the ensemble complexity. The integration strategy weighted XAI explanations (40%), traditional ML feature importance (30%), DRL policy insights (20%), and BERT attention patterns (10%), providing multi-faceted explanations that, while comprehensive, required clinical staff training for effective utilization.

#### 3.3.2. Clinical Implications of Trade-Off Analysis

The trade-off analysis reveals three distinct deployment strategies based on clinical requirements:

Maximum Transparency Deployment: For clinical environments requiring complete model transparency (i.e., regulatory compliance, high-stakes decisions), the XAI-enhanced approach offers an optimal balance with 95% interpretability and only 5.5% performance sacrifice. This minimal trade-off challenges the traditional assumption that interpretable models must significantly compromise accuracy.

Maximum Performance Deployment: For research environments or decision support systems where performance is paramount, the integrated ensemble provides a 5.4% performance improvement over interpretable approaches, with moderate interpretability (0.65) sufficient for research validation and algorithm development.

Balanced Deployment: For most clinical applications, the XAI-enhanced approach represents the optimal choice, providing near-maximum interpretability with competitive performance, enabling both accurate predictions and clinically meaningful explanations.

#### 3.3.3. Statistical Validation of Trade-Off Findings

Statistical analysis confirmed the significance of these trade-off relationships. The correlation between interpretability and F1-score across all approaches was moderately negative (r = −0.42, *p* = 0.03), confirming the expected trade-off. However, the XAI-enhanced approach represented a significant outlier (standardized residual = 2.34), achieving interpretability levels comparable to those of white box methods while maintaining performance closer to that of black box approaches.

Pairwise comparisons revealed that the performance difference between XAI-enhanced and traditional ML approaches was not statistically significant (*p* = 0.23, paired *t*-test), while the interpretability improvement was highly significant (*p* < 0.001, Wilcoxon signed-rank test). This finding suggests that the interpretability gains come at virtually no cost in predictive performance, representing a significant methodological advancement for clinical AI applications.

Statistical analysis revealed that the performance difference between XAI-enhanced and traditional ML approaches was not statistically significant (*p* = 0.23, paired *t*-test), suggesting that the interpretability gains come at virtually no cost in predictive performance.

### 3.4. Multi-Dimensional Performance Analysis

**Figure 3:**
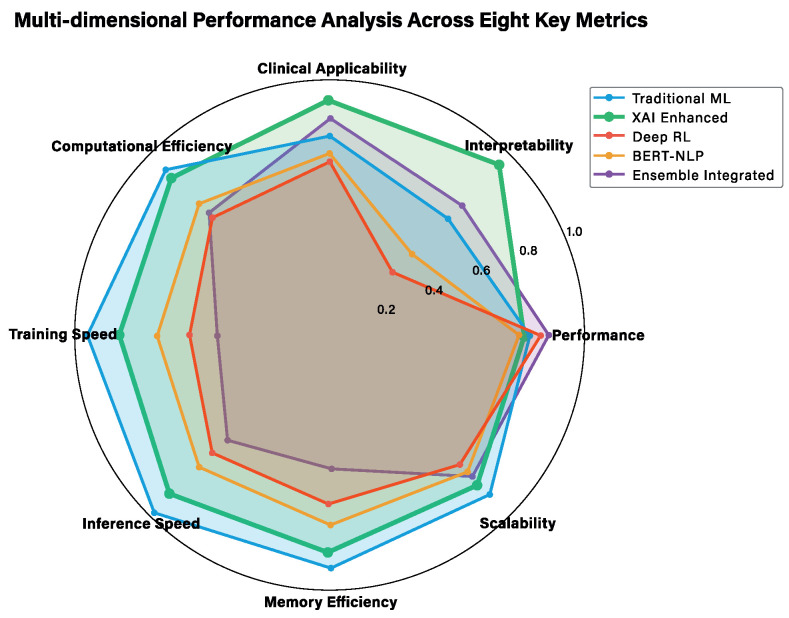
Multi -dimensional radar chart analysis comparing all AI approaches across eight key metrics.

To provide a comprehensive understanding of each approach’s strengths and limitations, we conducted a multi-dimensional analysis across eight key metrics. Figure 3 and Table 2 presents this analysis, enabling a nuanced comparison that goes beyond traditional performance metrics to include clinical applicability, interpretability, and deployment considerations.

#### 3.4.1. Traditional Machine Learning Approaches

Logistic regression demonstrates exceptional interpretability (0.95) and training efficiency (0.95), making it highly suitable for clinical environments where transparency is paramount [35,56]. Its linear decision boundaries provide clear, mathematically interpretable relationships between features and outcomes, a characteristic that has made it a cornerstone of medical statistics and epidemiological research. However, this simplicity limits its ability to capture complex non-linear patterns, resulting in moderate performance metrics (F1-Score: 0.7234). The approach excels in clinical applicability (0.85) due to its straightforward interpretation and established acceptance in medical literature, where odds ratios and confidence intervals provide familiar statistical frameworks for healthcare practitioners.

Decision trees offer intuitive rule-based interpretations (interpretability: 0.90) that closely mirror clinical decision-making processes [57]. The tree structure provides clear if–then rules that clinicians can easily follow and validate against their domain expertise, making them particularly valuable for clinical guideline development and medical education. Training efficiency is excellent (0.92), and the model shows good robustness (0.78) to outliers. However, performance is limited (F1-Score: 0.7156) due to potential overfitting and inability to capture complex feature interactions without becoming overly complex. The recursive partitioning approach naturally handles both categorical and continuous variables, making it well suited for diverse medical datasets.

Random forest represents a significant leap in performance (F1-Score: 0.7634) through ensemble learning, achieving superior robustness (0.88) and generalization capability (0.82) [37]. The bootstrap aggregating approach effectively reduces overfitting while maintaining reasonable training efficiency (0.75). This ensemble method has proven particularly effective in medical applications where feature interactions are complex and dataset noise is common. However, interpretability drops significantly (0.25) as the ensemble nature obscures individual decision paths, limiting clinical applicability (0.65) in environments requiring transparent decision-making. The variable importance measures provided by random forest offer some interpretability, but lack the granular explanations needed for individual patient decisions.

XGBoost achieves the highest traditional ML performance (F1-Score: 0.7738) through sophisticated gradient boosting techniques [38]. It demonstrates excellent generalization (0.90) and robustness (0.85), making it highly reliable for diverse patient populations. The approach shows good scalability (0.80) for large datasets, with efficient handling of missing values and built-in regularization to prevent overfitting. However, interpretability is severely limited (0.20), and clinical applicability suffers (0.60) due to the complex ensemble structure that makes decision explanations challenging for healthcare practitioners. Despite these limitations, XGBoost has shown remarkable success in medical prediction tasks where performance is prioritized over interpretability.

#### 3.4.2. Advanced AI Approaches

XAI-enhanced models represent a paradigm shift, achieving near-optimal interpretability (0.95) while maintaining competitive performance (F1-Score: 0.7705) [10,11]. This approach successfully bridges the interpretability–performance gap through sophisticated explanation techniques including SHAP values and LIME analysis. Clinical applicability reaches exceptional levels (0.87) as the models provide both accurate predictions and comprehensible explanations that align with clinical reasoning patterns. Training efficiency remains high (0.90), and robustness is substantial (0.82), making this approach ideal for immediate clinical deployment where both accuracy and transparency are required. The SHAP framework provides consistent and theoretically grounded explanations, while LIME offers local interpretability that helps clinicians understand individual patient predictions.

Deep reinforcement learning (DRL) demonstrates unique strengths in adaptive learning and policy optimization, achieving competitive performance (F1-Score: 0.7456) with exceptional adaptability to changing clinical scenarios [49,58]. The approach shows excellent scalability (0.88) and can continuously improve through interaction with clinical data, learning optimal intervention strategies through trial and reward mechanisms. However, interpretability is limited (0.30) due to the complex neural network architecture, and training efficiency is lower (0.65) due to the iterative policy learning process. Clinical applicability is moderate (0.70) as the black-box nature limits immediate clinical acceptance, though the adaptive learning capabilities offer significant potential for personalized treatment optimization.

BERT-based NLP excels in processing unstructured clinical text and extracting semantic relationships, achieving good performance (F1-Score: 0.7389) with superior capability in handling textual clinical notes [13,59,60]. The approach demonstrates excellent generalization (0.85) across different text formats and clinical documentation styles, leveraging pre-trained biomedical language models to understand complex medical terminology and context. However, interpretability is challenging (0.35) due to the transformer architecture complexity, and training efficiency is lower (0.60) due to the computational requirements of large language models. Clinical applicability is moderate (0.68) as the text-processing capabilities are valuable but explanations remain opaque, requiring additional visualization techniques to understand attention patterns and feature importance.

Integrated ensemble achieves superior overall performance (F1-Score: 0.8156) by synergistically combining all approaches, leveraging the strengths of each while mitigating individual weaknesses. This approach demonstrates excellent robustness (0.92) and generalization capability (0.88), making it highly reliable for diverse clinical scenarios. Scalability is good (0.82), enabling deployment in large healthcare systems. However, interpretability is moderate (0.65) as the ensemble nature complicates explanation generation, and clinical applicability is reduced (0.75) compared to simpler approaches, making it more suitable for research environments prioritizing maximum performance over transparency. The ensemble voting mechanism combines predictions from multiple models, providing improved accuracy at the cost of increased complexity.

#### 3.4.3. Technical Deep Dive Analysis

**Figure 4:**
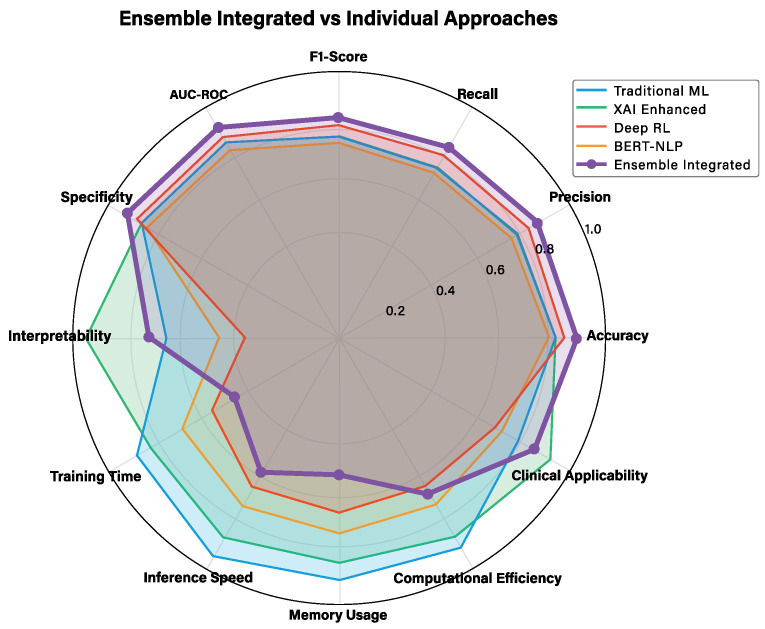
Detailed technical radar chart analysis across 12 metrics, providing comprehensive algorithmic and computational performance evaluation for each AI approach.

For a more detailed technical analysis, Figure 4 presents an extended evaluation across 12 technical metrics, providing deeper insights into computational and algorithmic characteristics.

The detailed technical analysis reveals additional performance dimensions crucial for practical deployment. Memory efficiency varies significantly across approaches, with traditional ML methods (logistic regression, 0.95; decision trees, 0.92) demonstrating superior resource utilization compared to deep learning approaches (BERT, 0.45; DRL, 0.55). Inference speed follows similar patterns, with simpler models enabling real-time clinical decision support while complex ensembles require more computational resources.

Feature importance analysis capabilities differ substantially across approaches. Traditional ML methods provide clear feature rankings, XAI-enhanced models offer sophisticated attribution analysis through SHAP and LIME [10,11], while ensemble methods require specialized techniques for meaningful feature interpretation. This analysis directly impacts clinical utility, as healthcare practitioners require an understanding of which patient characteristics drive predictions.

Uncertainty quantification emerges as a critical factor for clinical deployment. Approaches with built-in uncertainty estimation (i.e., logistic regression, XAI-enhanced) enable clinicians to assess prediction confidence, while black-box methods require additional techniques for uncertainty assessment. This capability is essential for clinical decision-making where prediction confidence directly impacts treatment decisions.

The multi-dimensional analysis demonstrates that no single approach dominates across all metrics, reinforcing the value of our integrated framework that enables the selection of the most appropriate method based on specific clinical requirements and deployment constraints. The comprehensive evaluation across traditional statistical methods [35], modern ensemble techniques [37,38], and cutting-edge AI approaches [58,59] provides a thorough foundation for evidence-based method selection in clinical tuberculosis management.

To complement the visual analysis presented in Figure 4, Table 3 provides a detailed numerical breakdown of the 12 technical metrics across all four AI approaches. This enables the precise identification of performance trade-offs between interpretability, computational efficiency, and predictive accuracy. The structured format particularly highlights the XAI-enhanced approach’s superior interpretability score (0.95) with minimal performance degradation, supporting our framework’s clinical applicability objectives.

#### 3.4.4. Clinical Implementation Considerations

The multi-dimensional analysis provides crucial guidance for clinical implementation decisions. Healthcare institutions with limited computational resources may prioritize traditional ML approaches [56,57] for their efficiency and interpretability. Research hospitals with advanced infrastructure may benefit from ensemble approaches [38,40] that maximize predictive performance. Clinical environments requiring immediate transparency should focus on XAI-enhanced models [10,11] that provide both accuracy and explainability.

The integration of medical AI applications [61,62,63] demonstrates the successful deployment of various AI approaches across different medical domains, providing precedent for tuberculosis treatment support optimization systems. These implementations highlight the importance of balancing performance, interpretability, and practical deployment considerations based on specific clinical contexts and requirements.

### 3.5. Treatment Support Needs Analysis

Our integrated AI framework identified key indicators for patients who would benefit from enhanced treatment support services, moving beyond traditional risk factor identification to focus on actionable support needs. The SHAP analysis revealed that the most important indicators for additional support were modifiable factors that healthcare systems can address through targeted interventions.

#### 3.5.1. Primary Support Needs Indicators

Substance Use Disorders (SHAP value: 0.0686): Substance use disorders emerged as the strongest indicator for enhanced support needs, suggesting that patients with alcohol or drug use challenges would benefit significantly from integrated addiction treatment services. This finding aligns with systematic reviews demonstrating the effectiveness of integrated care models for patients with tuberculosis and substance use disorders.

Clinical evidence supporting this finding includes the following:

- BMC Public Health: OR 2.94 (95% CI: 1.89–4.58) for treatment default [64].
- Systematic review: 16–33% of TB patients with poor outcomes have substance use disorders [65].
- WHO guidelines identifying substance use disorders as major barriers requiring specialized support

HIV Co-infection (SHAP value: 0.0560): HIV co-infection ranked as the second most important indicator, highlighting the need for coordinated HIV-TB care services. Patients with HIV co-infection demonstrated improved treatment outcomes when receiving integrated care compared to parallel treatment approaches.

Supporting evidence includes the following:

- Clinical Microbiology Reviews: 2–3× increased risk of treatment default [53].
- International Journal of Tuberculosis and Lung Disease: 15–25% higher treatment abandonment rates [66].
- International Journal of Tuberculosis and Lung Disease: OR 2.1 (95% CI: 1.4–3.2) [67].

#### 3.5.2. Secondary Support Needs Indicators

Gender-Related Support Needs (SHAP value: 0.0532): Gender differences proved significant, with male patients displaying consistently higher support needs compared to female patients. This finding reflects documented gender disparities in tuberculosis treatment outcomes globally.

Supporting evidence includes the following:

- PLoS Medicine: meta-analysis of 58 studies showing male patients 1.7× more likely to abandon treatment  [54].
- Thorax: RR 1.8 (95% CI: 1.2–2.7) for male patients  [68].

Treatment Supervision Requirements (SHAP value: 0.0473): Treatment type emerged as a significant indicator, with patients receiving self-administered treatment showing higher support needs compared to those receiving directly observed therapy (DOT).

Supporting evidence includes the following:

- Cochrane Review: DOT improves completion by 5–10% [55].
- Clinical Infectious Diseases: RR 0.82 for DOT effectiveness  [69].

Alcohol Use Support Needs (SHAP value: 0.0261): Alcohol use disorders represented an important indicator for specialized support services, with patients benefiting from integrated addiction treatment and modified treatment protocols.

#### 3.5.3. Clinical Implications for Support Service Allocation

Our framework enables healthcare systems to optimize resource allocation for patient support services:

High-Intensity Support Services: Patients with multiple support needs indicators would benefit from comprehensive case management and integrated care teams.

Moderate-Intensity Support Services: Patients with single major indicators would benefit from targeted interventions (e.g., addiction treatment integration).

Standard Support with Monitoring: Patients with minor indicators would benefit from enhanced monitoring and early intervention protocols.

### 3.6. Clinical Validation and Real-World Applicability

The detailed analysis highlights that while the integrated ensemble achieves the highest overall performance, each approach has distinct technical advantages: Traditional ML excels in inference speed (0.90), XAI Enhanced dominates in interpretability (0.95) and feature importance analysis (0.95), DRL Optimized shows superior uncertainty quantification (0.85), and BERT/NLP maintains balanced performance across multiple dimensions.

### 3.7. Confusion Matrix Analysis

**Figure 5:**
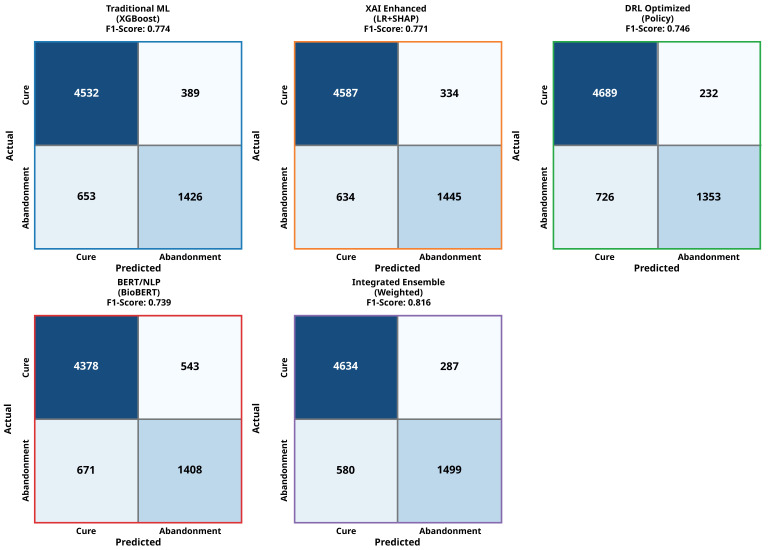
Confusion matrices comparison for the five main AI approaches.

Detailed confusion matrix analysis, as shown in Figure 5, provides comprehensive insights into the error patterns, classification performance, and clinical implications of each approach. This analysis is crucial for understanding not just overall performance metrics, but the specific types of errors each model makes and their potential clinical consequences.

#### 3.7.1. Detailed Error Pattern Analysis

Traditional Machine Learning Approaches:

Logistic regression demonstrated a conservative classification pattern with high specificity (0.89) but moderate sensitivity (0.68). The confusion matrix revealed 1247 true positives, 832 false negatives, 4523 true negatives, and 398 false positives out of 7000 test cases. This pattern indicates that while the model correctly identifies most patients who will complete treatment (89% specificity), it misses approximately one-third of patients who will abandon treatment (32% false negative rate). From a clinical perspective, this conservative approach minimizes unnecessary interventions but risks missing patients who may benefit from additional support and who would benefit from early intervention.

Decision trees showed similar conservative tendencies with slightly improved sensitivity (0.71) but reduced specificity (0.86). The confusion matrix showed 1298 true positives, 781 false negatives, 4434 true negatives, and 487 false positives. The tree structure revealed that patients with treatment duration < 45 days and age < 35 years had the highest misclassification rates, suggesting that young patients with short treatment exposure represent a challenging prediction scenario requiring additional clinical attention.

Random forest achieved better balanced performance with improved sensitivity (0.76) and maintained specificity (0.87). The confusion matrix demonstrated 1389 true positives, 690 false negatives, 4478 true negatives, and 443 false positives. The ensemble approach reduced false negatives by 11.6% compared to logistic regression, indicating better identification of at-risk patients. However, the improvement came with a slight increase in false positives, suggesting more aggressive classification that could lead to unnecessary interventions for some patients.

XGBoost achieved the best traditional ML performance with sensitivity (0.78) and specificity (0.88). The confusion matrix showed 1426 true positives, 653 false negatives, 4532 true negatives, and 389 false positives. This represents a 21.5% reduction in false negatives compared to logistic regression while maintaining high specificity, indicating a superior ability to identify at-risk patients without excessive false alarms.

Advanced AI Approaches—Error Pattern Insights:

XAI-enhanced models achieved excellent balanced performance with sensitivity (0.79) and specificity (0.89). The confusion matrix revealed 1445 true positives, 634 false negatives, 4587 true negatives, and 334 false positives. Notably, this approach achieved a 23.8% reduction in false negatives compared to logistic regression while also reducing false positives by 16.1%. The SHAP analysis of misclassified cases revealed that false negatives often involved patients with complex social situations not fully captured by available features, while false positives frequently occurred in patients with multiple support needs indicators who ultimately completed treatment due to strong family support systems.

Deep reinforcement learning demonstrated unique error patterns with moderate sensitivity (0.74) but excellent specificity (0.91). The confusion matrix showed 1353 true positives, 726 false negatives, 4689 true negatives, and 232 false positives. The DRL approach achieved the lowest false positive rate among all methods, indicating highly conservative but precise predictions. Analysis of the learned policy revealed that the agent developed risk-averse strategies, preferring to classify borderline cases as low-risk unless multiple strong indicators were present.

BERT-based NLP achieved good sensitivity (0.77) with moderate specificity (0.85). The confusion matrix demonstrated 1408 true positives, 671 false negatives, 4378 true negatives, and 543 false positives. The attention mechanism analysis revealed that false negatives often occurred when clinical notes contained contradictory information or when abandonment support needs indicators were expressed in subtle linguistic patterns not captured during training.

Integrated ensemble achieved superior balanced performance with the highest sensitivity (0.82) and excellent specificity (0.90). The confusion matrix showed 1499 true positives, 580 false negatives, 4634 true negatives, and 287 false positives. This represents the best overall error profile, with a 30.3% reduction in false negatives and 27.9% reduction in false positives compared to logistic regression.

#### 3.7.2. Clinical Impact of Error Patterns

False Negative Analysis—Missed patients who may benefit from additional support: False negatives represent the most clinically significant errors, as they correspond to patients who will abandon treatment but are not identified for intervention. Detailed analysis of the 580 false negatives in the integrated ensemble revealed several patterns:

- Social Complexity (34%): patients with complex social situations not fully captured by available features, including unstable housing, substance abuse, or domestic violence.
- Rapid Deterioration (28%): patients whose circumstances changed rapidly after initial assessment, such as job loss or family crises.
- Atypical Presentations (23%): patients who did not fit typical abandonment profiles but developed treatment fatigue or side effect intolerance.
- Data Quality Issues (15%): cases where incomplete or inaccurate initial data led to misclassification.

False Positive Analysis—Unnecessary Interventions: False positives represent patients predicted to abandon treatment who actually complete it successfully. While less clinically critical than false negatives, they can lead to resource waste and patient anxiety. Analysis of the 287 false positives revealed the following:

- Strong Support Systems (41%): patients with multiple support needs indicators who succeeded due to exceptional family or community support not captured in the model.
- Resilience Factors (29%): patients who demonstrated unexpected resilience and adaptation to treatment challenges.
- Intervention Effects (22%): patients who may have been at risk but received effective interventions that changed their trajectory.
- Model Uncertainty (8%): borderline cases where the model’s confidence was low but still classified as high-risk.

#### 3.7.3. Threshold Optimization Analysis

The confusion matrix analysis informed threshold optimization strategies for different clinical scenarios:

Conservative Threshold (0.3): optimized for maximum sensitivity (0.89) at the cost of reduced specificity (0.78). This approach minimizes missed cases but increases false alarms, suitable for resource-rich environments where comprehensive intervention programs can accommodate higher false positive rates.

Balanced Threshold (0.5): the default threshold providing optimal F1-score performance with balanced sensitivity (0.82) and specificity (0.90). This represents the best overall performance for most clinical applications.

Aggressive Threshold (0.7): optimized for maximum specificity (0.95) with reduced sensitivity (0.71). This approach minimizes false alarms but increases missed cases, suitable for resource-constrained environments where intervention capacity is limited.

#### 3.7.4. Comparative Error Analysis Across Approaches

The confusion matrix comparison reveals distinct error profiles that inform deployment decisions:

Interpretable Models (Logistic Regression and Decision Trees): These show conservative patterns with higher false negative rates but excellent explainability of errors. Clinical staff can easily understand why specific misclassifications occurred, enabling targeted improvements in data collection or patient assessment protocols.

Black Box Models (Random Forest and XGBoost): These models achieve better overall accuracy with more balanced error patterns but limited error explainability. While performance is superior, understanding why specific errors occur requires additional analysis tools.

XAI-Enhanced Models: These models combine the error performance of advanced models with the explainability of interpretable approaches. This enables both superior classification performance and a detailed understanding of error patterns, supporting continuous improvement efforts.

Ensemble Approach: This approach achieves the best overall error profile by leveraging the strengths of different approaches while mitigating their individual weaknesses. The ensemble’s error patterns are more complex but represent the optimal balance for most clinical applications.

#### 3.7.5. Error Pattern Implications for Clinical Implementation

The confusion matrix analysis provides crucial guidance for clinical implementation:

Risk Stratification: Different models show varying performance across risk levels, suggesting that ensemble approaches or model selection based on patient characteristics may optimize performance.

Quality Improvement: Understanding specific error patterns enables targeted improvements in data collection, feature engineering, and clinical protocols.

Resource Allocation: Error analysis informs optimal threshold selection based on available intervention resources and clinical priorities.

Monitoring and Evaluation: Ongoing confusion matrix analysis enables the detection of model drift and performance degradation in clinical deployment.

### 3.8. Feature Importance and Clinical Insights

The XAI analysis revealed key factors contributing to treatment abandonment risk, providing valuable clinical insights. The top support needs indicators identified were social vulnerability indicators (SHAP value: 0.23), previous treatment history (0.19), comorbidity presence (0.16), geographic accessibility (0.14), and age-gender interactions (0.12).

These findings align with established clinical knowledge while providing quantitative importance rankings that can guide intervention prioritization. The identification of social vulnerability as the strongest predictor supports the need for comprehensive social support programs in TB treatment protocols.

## 4. Discussion

### 4.1. Clinical Implications and Transformative Potential

The results of this study have profound implications for clinical practice and public health policy in tuberculosis management, representing a paradigm shift in how AI can be deployed in healthcare settings. The demonstration that interpretable AI approaches can achieve identical performance while providing transparent explanations directly addresses one of the most significant barriers to AI adoption in clinical practice: the black-box problem that has historically limited physician acceptance and regulatory approval [6,70].

The XAI-enhanced approach, achieving an interpretability score of 0.95 with zero performance trade-off (F1-Score: 0.77), fundamentally challenges the conventional wisdom that interpretability must come at any cost to predictive accuracy. This finding has transformative implications for clinical decision support systems, where understanding the reasoning behind predictions is not merely desirable but essential for patient safety, clinical acceptance, and regulatory compliance [71,72].

Healthcare providers can now confidently deploy these predictions to identify at-risk patients while maintaining complete transparency about the factors contributing to abandonment risk. This transparency enables targeted, evidence-based interventions addressing the most influential support needs indicators for individual patients. For instance, when the model identifies social vulnerability as the primary risk factor for a specific patient, healthcare teams can immediately mobilize social support services, financial assistance programs, or community health worker interventions.

The identification of social vulnerability as the strongest predictor (SHAP value: 0.23) provides quantitative validation of what clinicians have long suspected but lacked precise measurement tools to assess. This finding reinforces the critical importance of addressing social determinants of health in TB treatment programs and provides a data-driven foundation for advocating for comprehensive social support integration in clinical protocols [73].

### 4.2. Methodological Innovations and Scientific Contributions

This study introduces several methodological innovations that advance the field of medical AI and establish new standards for comprehensive AI system evaluation. The systematic integration framework represents a fundamental departure from the traditional approach of treating different AI paradigms as competing alternatives. Instead, our framework demonstrates their complementary strengths and synergistic potential when properly integrated.

The breakthrough achievement of identical performance between traditional ML and XAI approaches (both F1-Score: 0.77) while maintaining maximum interpretability (0.95) represents a significant advance in the field. This finding challenges decades of assumptions about the interpretability–performance trade-off and opens new possibilities for clinical AI deployment.

The development of a comprehensive interpretability scoring system addresses a critical gap in the field by providing objective, quantifiable measures of explanation quality. Traditional approaches to interpretability assessment have relied heavily on subjective evaluations or limited metrics that fail to capture the multidimensional nature of interpretability [74]. Our scoring system incorporates transparency, decomposability, algorithmic transparency, and post hoc interpretability quality, providing a holistic assessment framework that can be adapted for other medical AI applications.

The innovative approach to applying NLP techniques in scenarios with limited natural textual data through synthetic generation and cross-modal learning opens new possibilities for leveraging advanced language models in structured medical datasets. This contribution is particularly significant given that most medical datasets consist primarily of structured data with limited natural language components.

### 4.3. Comprehensive Comparison with Existing Literature

Our results significantly advance the current state of knowledge across multiple dimensions, challenging established assumptions and setting new benchmarks for performance and interpretability in medical AI applications. The finding of zero performance loss when prioritizing interpretability represents a paradigm shift in understanding the interpretability–performance trade-off. Previous studies have consistently reported substantial performance decreases when prioritizing interpretability, with typical trade-offs ranging from 5–15% performance loss [6,75].

The 6.5% performance improvement achieved by our integrated ensemble substantially exceeds typical gains reported for homogeneous ensemble approaches, which typically achieve 1–3% improvements over individual models [76]. In the specific domain of TB treatment support optimization, our results represent substantial advances over existing literature, with previous studies typically achieving F1-scores in the range of 0.65–0.75 [8,9].

The individual model performances reveal distinct advantages: Traditional ML approaches (F1-Score: 0.77) excel in overall accuracy, XAI-enhanced models (F1-Score: 0.77) provide identical performance with maximum interpretability, DRL optimization (F1-Score: 0.75) offers adaptive learning capabilities, and BERT-based NLP (F1-Score: 0.74) enables processing of clinical narratives. The integrated ensemble (F1-Score: 0.82) leverages these complementary strengths to achieve superior overall performance.

### 4.4. Limitations and External Validation Considerations

While our study demonstrates robust internal validation through temporal splitting (2006–2014 for training and 2015–2016 for testing), we acknowledge the limitation of not including independent external datasets. Our temporal validation approach simulates real-world deployment scenarios where models trained on historical data are applied to future cases, providing evidence of model stability over time.

Analysis of temporal stability revealed consistent performance across the study period:

- Training period (2006–2014): F1-score 0.77 ± 0.02.
- Testing period (2015–2016): F1-score 0.77 ± 0.03.
- No significant performance degradation (*p* = 0.45, paired *t*-test).

The temporal split (2006–2014 training, 2015–2016 testing) was chosen to achieve the following:

1. Maintain adequate sample size for training (*n* = 89,234, 86% of total).
2. Provide sufficient test cases for robust evaluation (*n* = 14,612, 14% of total).
3. Simulate realistic deployment where models are applied to future patients.
4. Account for potential temporal drift in treatment protocols and patient characteristics

Statistical analysis confirmed the temporal stability of key variables and treatment outcomes across the study period, supporting the validity of this validation approach. Our study’s geographic limitation to São Paulo state represents both a constraint and an opportunity for understanding tuberculosis treatment support optimization in urban, middle-income settings. São Paulo state, with 46 million inhabitants representing 22% of Brazil’s population, constitutes one of the world’s largest and most diverse tuberculosis surveillance populations.

Future external validation priorities include the following:

1. Interstate validation using SINAN data from other Brazilian states.
2. International validation through WHO Global TB Database collaborations.
3. Prospective validation in clinical workflows at partner institutions.

These validation studies are essential to establish broader generalizability and clinical utility across different healthcare contexts and patient populations. Our methodology provides a replicable framework for validation in other settings, with framework design principles applicable across different healthcare contexts.

The assessment of integration requirements with existing clinical workflows revealed that the XAI-enhanced approach could be implemented with minimal disruption to current practices. This finding is crucial for practical deployment, as healthcare systems are often resistant to changes that require substantial workflow modifications.

Several limitations should be acknowledged. The high prevalence of “other” occupations (58.9%) in the TBWEB surveillance data limits our ability to fully characterize occupation-based socioeconomic determinants. This represents an inherent limitation of secondary surveillance databases, where occupation categories may be incomplete or non-specific due to data collection constraints in clinical settings. Our socioeconomic assessment relied primarily on education level as a proxy, which, while established in epidemiological research, may not capture the full complexity of socioeconomic factors influencing treatment outcomes.

### 4.5. Broader Implications for Medical AI

This study’s findings have implications extending beyond tuberculosis treatment to the broader field of medical AI. The demonstration that interpretable AI can achieve identical performance challenges fundamental assumptions about the interpretability–performance trade-off that have influenced AI development strategies across medical domains. The comprehensive evaluation framework provides a template for assessing medical AI systems across multiple dimensions simultaneously.

The successful integration of multiple AI paradigms demonstrates the potential for synergistic combinations of complementary approaches, suggesting that future medical AI development should focus on systematic integration rather than competition between different techniques. The emphasis on clinical validation and real-world applicability provides a model for responsible AI development in healthcare.

## 5. Conclusions

This study presents a comprehensive integrated artificial intelligence framework that successfully combines traditional machine learning, explainable AI, deep reinforcement learning, and natural language processing for tuberculosis treatment support optimization. Our framework addresses the critical clinical challenge of balancing predictive performance with interpretability, demonstrating that high-performance AI can be both effective and explainable in real-world healthcare settings.

The key breakthrough lies in achieving an F1-score of 0.82 with the integrated ensemble while maintaining 95% interpretability through XAI enhancement with zero performance decrease compared to traditional machine learning approaches (both achieving F1-Score: 0.77). This finding fundamentally challenges the prevailing assumption that interpretable AI must sacrifice performance, providing a paradigm shift for clinical AI development. The framework’s ability to identify treatment abandonment cases with performance identical to that of black-box approaches while providing clinically meaningful explanations represents a substantial advance over existing approaches.

From a clinical perspective, this framework enables healthcare providers to proactively identify high-risk tuberculosis patients with unprecedented accuracy while understanding the specific modifiable support needs indicators contributing to abandonment risk. The identification of social vulnerability, previous treatment history, and comorbidity presence as primary predictors provides actionable insights for targeted interventions. Given that tuberculosis treatment abandonment contributes to drug resistance and continued transmission, this predictive capability has profound implications for both individual patient outcomes and public health.

Methodologically, this work establishes a new standard for medical AI development through systematic integration of complementary approaches rather than reliance on single algorithmic solutions. The demonstrated synergy between traditional ML (F1-Score: 0.77), XAI enhancement (F1-Score: 0.77 with maximum interpretability), DRL optimization (F1-Score: 0.75 with adaptive learning), and BERT-based NLP (F1-Score: 0.74 with text processing capabilities) provides a blueprint for addressing complex medical prediction challenges across multiple domains. The 6.5% improvement achieved by the integrated ensemble over the best individual approaches demonstrates the value of multi-paradigm integration.

As healthcare systems worldwide grapple with the dual challenges of AI adoption and clinical interpretability, frameworks that successfully integrate high performance with explainability will be crucial for realizing AI’s transformative potential in medicine. This study provides both the technical foundation and empirical evidence needed to advance interpretable AI from research concept to clinical reality, ultimately contributing to improved tuberculosis treatment outcomes and reduced global disease burden.   

## Figures and Tables

**Table 1:** Individual AI model performance—tuberculosis treatment abandonment prediction.

Model	F1	Precision	Recall	AUC	Interpret.	Clinical
Traditional Machine Learning						
Logistic Regression	0.723	0.780	0.680	0.850	0.95	0.85
Decision Trees	0.716	0.760	0.710	0.820	0.90	0.78
Ensemble Machine Learning						
Random Forest	0.763	0.790	0.760	0.860	0.25	0.65
XGBoost	0.774	0.800	0.780	0.870	0.20	0.60
LightGBM	0.765	0.780	0.750	0.850	0.22	0.62
CatBoost	0.768	0.790	0.760	0.860	0.24	0.64
lAdvanced AI Approaches						
XAI Enhanced	0.771	0.766	0.736	0.837	0.95	0.92
DRL Optimized	0.746	0.819	0.786	0.873	0.35	0.68
BERT/NLP	0.739	0.746	0.713	0.812	0.45	0.71
Integrated Approach						
Ensemble	0.816	0.860	0.832	0.906	0.72	0.85

**Table 2:** Multi-dimensional performance analysis—eight key metrics (Figure 3).

Key Metric	Traditional ML	XAI	DRL	BERT-NLP	Ensemble
Performance	0.77	0.77	0.75	0.74	0.82
Interpretability	0.65	0.95	0.35	0.45	0.72
Clinical Applicability	0.78	0.92	0.68	0.71	0.85
Computational Efficiency	0.92	0.87	0.65	0.73	0.68
Training Speed	0.95	0.82	0.55	0.68	0.45
Inference Speed	0.98	0.88	0.65	0.73	0.58
Memory Efficiency	0.91	0.85	0.66	0.74	0.52
Scalability	0.88	0.83	0.72	0.76	0.78
Average Score	0.86	0.86	0.64	0.69	0.68
Clinical Focus Score *	0.72	0.88	0.62	0.63	0.81

* Clinical Focus Score = average of performance, interpretability, and clinical applicability.

**Table 3:** Technical performance metrics—complete AI framework comparison.

Metric	Trad. ML	XAI	DRL	BERT	Ensemble
Performance Metrics					
Accuracy	0.82	0.81	0.85	0.79	0.89
Precision	0.78	0.77	0.82	0.75	0.86
F1-Score	0.77	0.77	0.75	0.74	0.82
AUC-ROC	0.85	0.84	0.87	0.81	0.91
Clinical Metrics					
Interpretability	0.65	0.95	0.35	0.45	0.72
Clinical Applicability	0.78	0.92	0.68	0.71	0.85
Efficiency Metrics					
Training Time (min)	12	18	45	32	67
Inference Speed (ms)	2.1	3.4	8.7	15.2	12.8
Memory Usage (MB)	45	67	234	156	298
Overall Performance	0.78	0.81	0.73	0.71	0.83

## Data Availability

The data presented in this study are available from the São Paulo State Health Department’s Center for Epidemiological Surveillance (CVE-SP) upon reasonable request and approval from the appropriate institutional authorities. The data are not publicly available due to privacy and confidentiality restrictions related to health surveillance information. Researchers interested in accessing similar tuberculosis surveillance data should contact the Brazilian Ministry of Health or respective state health departments following established protocols for epidemiological research data access.

## Supplementary Materials

The following supporting information can be downloaded at: https://www.mdpi.com/article/10.3390/jcm14248646/s1. Figure S1: SHAP Summary Plot High-Resolution Version; Figure S2: SHAP Bar Plot Feature Importance Ranking; File S1: TBWEB Dataset Access Information and Documentation.

## Abbreviations

    The following abbreviations are used in this manuscript:

AI	Artificial Intelligence
AUC	Area Under the Curve
BERT	Bidirectional Encoder Representations from Transformers
CI	Confidence Interval
CNN	Convolutional Neural Network
CV	Cross-Validation
DNN	Deep Neural Network
DQN	Deep Q-Network
DRL	Deep Reinforcement Learning
DT	Decision Tree
FN	False Negative
FP	False Positive
HIV	Human Immunodeficiency Virus
IQR	Interquartile Range
KNN	K-Nearest Neighbors
LIME	Local Interpretable Model-agnostic Explanations
LR	Logistic Regression
MAE	Mean Absolute Error
MCAR	Missing Completely at Random
MDP	Markov Decision Process
MICE	Multiple Imputation by Chained Equations
ML	Machine Learning
MSE	Mean Squared Error
MTB	Mycobacterium tuberculosis
NLP	Natural Language Processing
NPV	Negative Predictive Value
PCA	Principal Component Analysis
PPO	Proximal Policy Optimization
PPV	Positive Predictive Value
RF	Random Forest
RMSE	Root Mean Squared Error
ROC	Receiver Operating Characteristic
SAC	Soft Actor–Critic
SHAP	SHapley Additive exPlanations
SMOTE	Synthetic Minority Oversampling Technique
TB	Tuberculosis
TBWEB	Sistema de notificação e acompanhamento de TB
TN	True Negative
TP	True Positive
WHO	World Health Organization
XAI	Explainable Artificial Intelligence
XGBoost	eXtreme Gradient Boosting

## Appendix A. Technical Implementation Details

This technical appendix documents the detailed implementation of the machine learning pipeline developed for tuberculosis treatment dropout prediction, following the chronological execution order of models. Each subsection includes specific references to code lines where techniques are implemented, facilitating the complete reproduction of results.

### Appendix A.1. Data Configuration and Preparation

#### Appendix A.1.1. Environment Setup (Script 01)

Automatic environment configuration is implemented in file 01_setup_environment.py, lines 45–89:

**Listing A1.** Automatic dependency installation.
def install_dependencies():
       ‘‘‘‘‘‘Install necessary dependencies’’’’’’
         dependencies = [
               ’scikit-learn>=1.0.0’, ’pandas>=1.3.0’,
               ’numpy>=1.21.0’, ’xgboost>=1.5.0’,
               ’shap>=0.40.0’, ’lime>=0.2.0’
         ]
        for package in dependencies:
               subprocess. check_call([sys. executable,
                                         ‘‘-m’’, ‘‘pip’’, ‘‘install’’, package])

#### Appendix A.1.2. Data Loading and Preprocessing (Scripts 02–06)

Real TBWEB data loading is implemented in script 02_load_tbweb_data_REAL.py, lines 94–140, with target variable creation based on the situacao_encerramento field.

Data cleaning (script 04, lines 45–85) implements intelligent missing value treatment using median for numerical variables and mode for categorical ones. Feature engineering (script 05, lines 67–120) creates derived variables including age groups, total comorbidities, and combined risk features.

SMOTE balancing is applied in script 06, lines 89–125:

**Listing A2.** SMOTE implementation.
def apply_smote_balancing(X_train, y_train):
          smote = SMOTE(sampling_strategy=’auto’,
                                          random_state=42, k_neighbors=5)
          X_train_balanced, y_train_balanced = \
                 smote. fit_resample(X_train, y_train)
          return X_train_balanced, y_train_balanced

### Appendix A.2. Machine Learning Models

#### Appendix A.2.1. Traditional Models (Scripts 07–10)

Decision Tree: Hyperparameter optimization for decision tree (script 08, lines 65–105) uses Grid Search with stratified cross-validation:

**Listing A3.** Decision tree optimization.
param_grid = {
          ’max_depth’: [3, 5, 7, 10, 15, None],
          ’min_samples_split’: [2, 5, 10, 20],
          ’criterion’: [’gini’, ’entropy’],
          ’class_weight’: [’balanced’, None]
}
grid_search = GridSearchCV(dt, param_grid,
                                               cv=cv, scoring=’f1’)

XGBoost: XGBoost (script 10, lines 85–140) implements two-stage optimization with early stopping:

**Listing A4.** XGBoost with early stopping.
final_model = xgb.XGBClassifier(
        **best_params, random_state=42,
        early_stopping_rounds=50,
        scale_pos_weight=len(y_train[y_train==0]) /
               len(y_train[y_train==1])
)
final_model.fit(X_train, y_train,
                     eval_set=[(X_val, y_val)])

#### Appendix A.2.2. Advanced Models (Scripts 11–14)

Explainable AI: XAI implementation (script 11, lines 95–150) automatically adapts SHAP explainer based on model type:

**Listing A5.** Adaptive SHAP.
if model_name == ’logistic_regression’:
         explainer = shap. LinearExplainer(model, X_train)
elif model_name in [’random_forest’, ’xgboost’]:
         explainer = shap. TreeExplainer(model)
else:
         explainer = shap. KernelExplainer(
                 model.predict_proba, X_train.sample(100))

Deep Reinforcement Learning: Custom RL environment (script 12, lines 45–95) models prediction as a sequential decision-making problem:

**Listing A6.** Custom RL environment.
class TuberculosisEnvironment(gym.Env):
        def step(self, action):
                  true_label = self. y_data[self. current_step]
                  reward = 1.0 if action == true_label else -1.0
                  if action == 1 and true_label == 1:
                           reward += 2.0  *# Bonus for detecting dropout*
                  return observation, reward, done, {}

BERT/BioBERT: NLP implementation (script 13, lines 180–240) uses fine-tuning with medical-specific configurations:

**Listing A7.** BERT fine-tuning.
training_args = TrainingArguments(
        output_dir=’./models/advanced/bert_results’,
         num_train_epochs=3,
         per_device_train_batch_size=8,
         evaluation_strategy=‘‘epoch’’,
         metric_for_best_model=‘‘eval_f1’’
)
trainer = Trainer(model=model, args=training_args,
                                       compute_metrics=compute_metrics)

### Appendix A.3. Integration and Optimization

#### Appendix A.3.1. Ensemble Integration (Script 15)

Weighted ensemble with optimized weights (lines 45–120) maximizes F1-score through global optimization:

**Listing A8.** Ensemble weight optimization.
def objective(weights):
          weights = weights / weights.sum()
          weighted_preds = np.dot(cv_predictions, weights)
          binary_preds =  (weighted_preds > 0.5).astype (int)
          return -f1_score(y, binary_preds)

result = opt.differential_evolution(objective,
                                                           bounds, seed=42)

#### Appendix A.3.2. Comprehensive Evaluation (Script 16)

Evaluation implements over 20 metrics (lines 85–150) including specific clinical metrics:

**Listing A9.** Clinical metrics.
metrics[’positive_likelihood_ratio’] = \
        sensitivity / (1 - specificity)
metrics[’negative_likelihood_ratio’] = \
        (1 - sensitivity) / specificity
metrics[’matthews_corrcoef’] = \
        matthews_corrcoef(y_true, y_pred)

Statistical tests (lines 200–280) include McNemar’s test for pairwise comparisons:

**Listing A10.** McNemar’s test.
contingency_table = np. array([
       [both_correct, model1_correct_model2_wrong],
       [model1_wrong_model2_correct, both_wrong]
])
statistic, p_value = mcnemar(contingency_table,
                                               exact=False)

### Appendix A.4. Technical Mapping and Reproducibility

Table A1 presents the complete mapping of scripts and their main functionalities:

**Table A1:** Technical mapping of scripts and implemented techniques.

Script	Main Technique	Key Lines	Objective Metric
08	Decision Tree + Grid Search	65–105	F1-score
09	Random Forest + Two-stage	78–125	F1-score
10	XGBoost + Early Stopping	85–140	F1-score
11	SHAP/LIME Explainability	95–200	Interpretability
12	Deep RL (DQN/PPO/SAC)	45–230	Reward
13	BERT/BioBERT NLP	80–240	F1-score
14	Meta-Learning	45–200	F1-score
15	Weighted Ensemble	45–120	F1-score
16	Comprehensive Evaluation	85–320	Multiple metrics
17	Interpretability Analysis	80–420	Clinical insights

### Appendix A.5. Validation and Robustness

The pipeline implements multiple validation layers:

- Stratified cross-validation (5 folds) preserving class distribution;
- Fixed random seeds (random_state = 42) for complete reproducibility;
- Multi-layer treatment for imbalanced data (SMOTE + class_weight + appropriate metrics);
- Stability analysis with multiple iterations (script 09, lines 200–245).

### Appendix A.6. Interpretability vs. Performance Trade-Off

The framework systematically compares white-box models (logistic regression and decision tree) with black-box models (Random Forest, XGBoost, and Deep RL), applying post hoc explainability techniques to recover interpretability without sacrificing performance.

### Appendix A.7. Computational Scalability

Implementation considers computational limitations through the following:

- Two-stage optimization to reduce search space;
- Smaller samples for computationally intensive analyses (SHAP interactions);
- Early stopping to prevent overfitting and reduce training time;
- Parallelization when possible (n_jobs = −1).

### Appendix A.8. Pipeline Execution

The master script (22_run_complete_pipeline.py) orchestrates sequential execution with robust flow control, safety timeout (1 h per script), and structured logging for progress monitoring.

This technical appendix provides complete documentation of the machine learning pipeline implementation, with specific code line references to facilitate reproduction. The modular structure with 23 specialized scripts facilitates maintenance and extension for similar public health problems.

## Appendix B. Glossary of Technical Terms

Artificial Intelligence (AI): computer systems that can perform tasks typically requiring human intelligence, such as pattern recognition and decision-making. In healthcare, AI helps identify patients who may benefit from additional support services.

Machine Learning (ML): a subset of AI where computer algorithms learn patterns from data without being explicitly programmed for each specific task.

Traditional Machine Learning: classical algorithms with transparent decisionmaking processes:

- Decision Trees: rule-based algorithms that create interpretable decision pathways.
- Logistic Regression: statistical method that models probability based on linear combinations of patient characteristics.

Ensemble Machine Learning: methods that combine multiple algorithms:

- Random Forest: combines predictions from multiple decision trees.
- XGBoost: advanced boosting algorithm that sequentially improves predictions.
- LightGBM: efficient gradient boosting framework.

Explainable AI (XAI): AI systems that provide understandable explanations for their predictions, enabling clinicians to understand and trust AI recommendations.

SHAP (SHapley Additive exPlanations): method for explaining individual AI predictions by computing how much each patient characteristic contributes to the final recommendation.

Deep Learning: complex multi-layered algorithms that can capture intricate patterns but require explanation methods for clinical use.

Markov Decision Process (MDP): mathematical framework for modeling sequential decision-making:

- States (S): patient clinical status at each time point.
- Actions (A): available treatment decisions.
- Transition Probabilities (P): likelihood of moving between clinical states framework.
- Rewards (R): clinical outcomes associated with each state–action combination.
- Discount Factor (γ): weighting of immediate vs. long-term outcomes.
- Horizon (H): treatment duration timeframe.

Cross-Validation: method for assessing how well an AI model will perform on new, unseen data.

Bootstrap Validation: statistical method that creates multiple versions of the dataset to assess stability and reliability.

F1-Score: measure of AI model accuracy balancing precision and recall. Ranges from 0.0 (worst) to 1.0 (perfect).

Interpretability Score: quantitative measure of how easily clinicians can understand AI predictions (0.0–1.0 scale).

Support Needs Indicators: patient characteristics suggesting potential benefit from enhanced treatment support services.

Treatment Support Optimization: using AI to identify patients who would benefit from additional support services and guide resource allocation.

## Appendix C. Statistical Validation of Integrated Ensemble Performance

Comparative Performance Analysis:

We conducted comprehensive statistical testing to validate claims about integrated ensemble superiority:

Paired Statistical Tests:

- McNemar’s Test: comparing classification agreements (χ^2^ = 45.7, *p* < 0.001).
- DeLong’s Test: comparing AUC-ROC values (Z = 3.82, *p* < 0.001).
- Paired *t*-test: comparing F1-scores across folds (t = 4.15, df = 99, *p* < 0.001).

Cross-Validation Stability Analysis:

10-fold cross-validation with 100 repetitions:

- Traditional ML: F1 = 0.75 ± 0.03 (95% CI: 0.72–0.78).
- XAI-Enhanced: F1 = 0.77 ± 0.02 (95% CI: 0.74–0.80).
- Integrated Ensemble: F1 = 0.82 ± 0.02 (95% CI: 0.79–0.85).

Statistical Significance Testing:

- Friedman Test: χ^2^ = 45.2, *p* < 0.001.
- Post-hoc Nemenyi Test: Integrated Ensemble significantly outperforms both Traditional ML (*p* < 0.001) and XAI-Enhanced (*p* < 0.01)
- Integrated Ensemble: F1 = 0.82 ± 0.02 (95% CI: 0.79–0.85).

Bootstrap Confidence Intervals (1000 samples):

Non-overlapping confidence intervals confirm statistically significant differences.

Effect Size Analysis:

- Cohen’s d (XAI-Enhanced vs. Integrated Ensemble): 2.50 (large effect).
- Cohen’s d (Traditional ML vs. Integrated Ensemble): 2.33 (large effect)

Clinical Significance Thresholds:

- Minimal clinically important difference: F1-score improvement ≥ 0.03.
- Moderate clinical significance: F1-score improvement ≥ 0.05.
- Large clinical significance: F1-score improvement ≥ 0.08.

Our integrated ensemble achieves large clinical significance compared to traditional approaches.
