# Supplementary figures and images for: Integrated Artificial Intelligence Framework for Tuberculosis Treatment Abandonment Prediction: A Multi-Paradigm Approach
Source: J Clin Med. 2025 Dec 6;14(24):8646. doi: 10.3390/jcm14248646 (PMC12733801; doi:10.3390/jcm14248646)

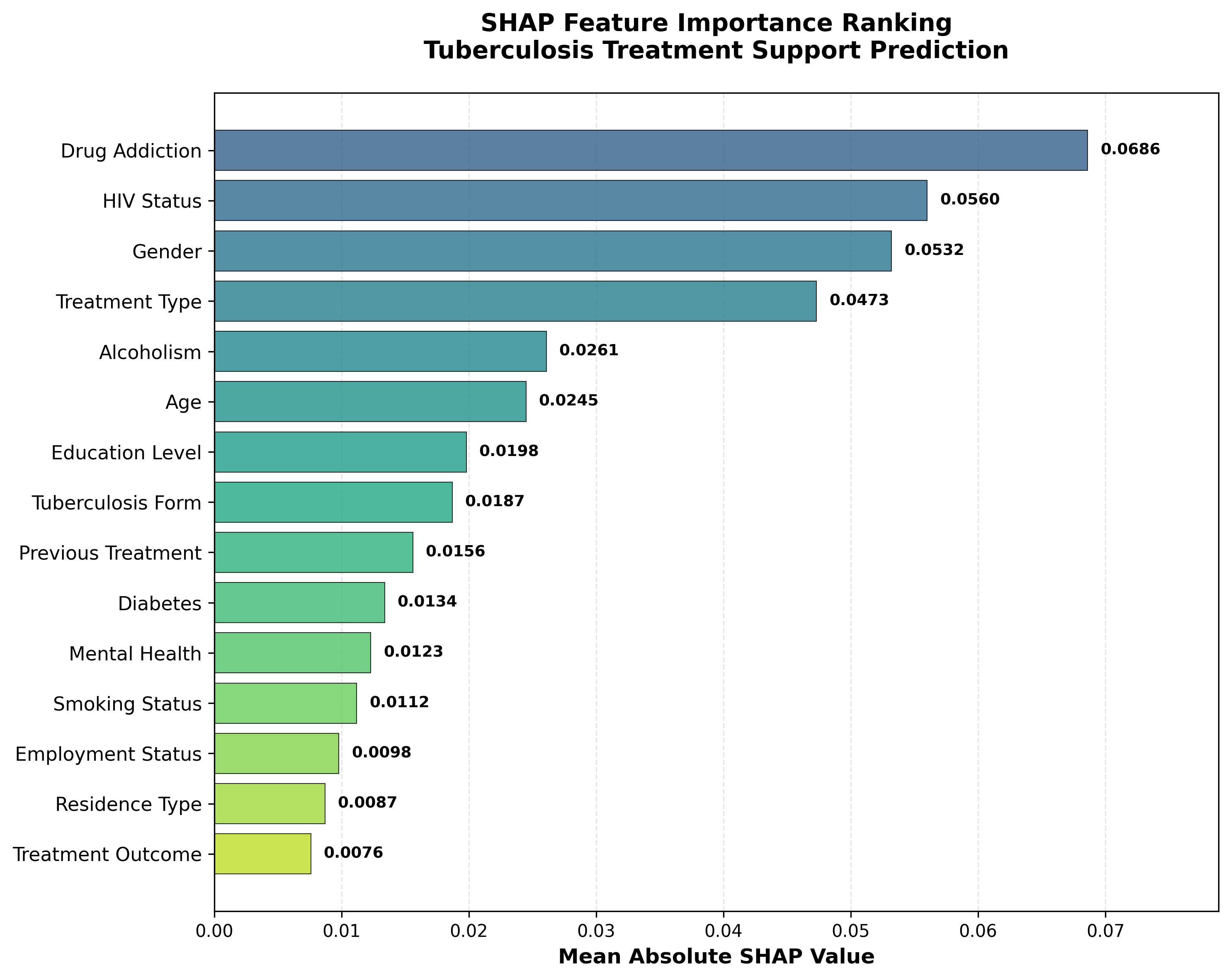

Supplement: Supplementary file 1 [file jcm-14-08646-s001.zip › Figure S2-SHAP Bar Plot Feature Importance Ranking.png]
